# Activation of GSK-3β and Caspase-3 Occurs in Nigral Dopamine Neurons during the Development of Apoptosis Activated by a Striatal Injection of 6-Hydroxydopamine

**DOI:** 10.1371/journal.pone.0070951

**Published:** 2013-08-05

**Authors:** Daniel Hernandez-Baltazar, Maria E. Mendoza-Garrido, Daniel Martinez-Fong

**Affiliations:** 1 Departamento de Fisiología, Biofísica y Neurociencias, Centro de Investigación y de Estudios Avanzados del Instituto Politécnico Nacional, México, D.F., México; 2 Programa de Nanociencias y Nanotecnología, Centro de Investigación y de Estudios Avanzados del Instituto Politécnico Nacional, México, D.F., México; National Institutes of Health, United States of America

## Abstract

The 6-Hydroxydopamine (6-OHDA) rat model of Parkinson's disease is essential for a better understanding of the pathological processes underlying the human disease and for the evaluation of promising therapeutic interventions. This work evaluated whether a single striatal injection of 6-OHDA causes progressive apoptosis of dopamine (DA) neurons and activation of glycogen synthase kinase 3β (GSK-3β) and caspase-3 in the substantia nigra compacta (SNc). The loss of DA neurons was shown by three neuron markers; tyrosine hydroxylase (TH), NeuN, and β-III tubulin. Apoptosis activation was determined using Apostain and immunostaining against cleaved caspase-3 and GSK-3β pY216. We also explored the possibility that cleaved caspase-3 is produced by microglia and astrocytes. Our results showed that the 6-OHDA caused loss of nigral TH(+) cells, progressing mainly in rostrocaudal and lateromedial directions. In the neostriatum, a severe loss of TH(+) terminals occurred from day 3 after lesion. The disappearance of TH(+) cells was associated with a decrease in NeuN and β-III tubulin immunoreactivity and an increase in Apostain, cleaved caspase-3, and GSK-3β pY216 in the SNc. Apostain immunoreactivity was observed from days 3 to 21 postlesion. Increased levels of caspase-3 immunoreactivity in TH(+) cells were detected from days 1 to 15, and the levels then decreased to day 30 postlesion. The cleaved caspase-3 also collocated with microglia and astrocytes indicating its participation in glial activation. Our results suggest that caspase-3 and GSK-3β pY216 activation might participate in the DA cell death and that the active caspase-3 might also participate in the neuroinflammation caused by the striatal 6-OHDA injection.

## Introduction

The unilateral 6-hydroxydopamine (6-OHDA) lesion of the nigrostriatal dopamine (DA) system in the rat has become an essential model for the understanding of the neuropathology of Parkinson’s disease (PD) [1]–[5], the pharmacological characterization of new antiparkinsonian drugs [6], and the evaluation of promising therapies for PD [7]–[9]. The 6-OHDA rat model does not replicate the abnormal protein aggregation of the familial PD [10], yet such a model certainly reproduces the oxidative damage [11], [12] and neuroinflammation [13] that occur in both familial and idiopathic forms of PD [14]. Though 6-OHDA has been used since the early 1960s [15], the molecular and cellular mechanisms of 6-OHDA cytotoxicity in the DA nigrostriatal system have not been fully characterized. Elucidation of those mechanisms is of critical importance to gain an insight into the mechanisms of neurotrophic factor therapy and those of drugs targeting the cell-death signaling pathway [16], [17]. These approaches are aimed to be used for the treatment of PD in the near future.

The notion that the 6-OHDA intrastriatal injection causes death of DA neurons in the substantia nigra compacta (SNc) is mainly supported by the decrease in the number of tyrosine hydroxylase (TH) immunoreactive cells [1], [3], [18], [19]. However, such a decrease might reflect a loss of phenotype (cells are present but no longer express TH) rather than the damage to cells and terminals. Some studies have suggested apoptosis in the SNc using silver staining or the terminal deoxynucleotidyl transferase dUTP nick-end labeling (TUNEL) assay after a 6-OHDA striatal injection [19]–[22]. Nonetheless, these studies did not accompany the TUNEL assays with other apoptosis markers such as caspases and key proteins in the signal transduction pathways of caspase activation to soundly support the participation of apoptosis in the effect of 6-OHDA. In addition, TUNEL also recognizes necrotic cells and thus its results are not conclusive of apoptosis participation, mainly when the studies *in vitro* have shown that 6-OHDA causes necrosis at the dosage commonly used *in vivo* [23], [24]. Most studies *in vivo* have shown the expression of cleaved caspase-3, the active form of this caspase [25], to show apoptosis in different models of cell death [26]–[28]. However, the participation of caspase-3 in apoptosis of the nigral DA neurons after a 6-OHDA striatal injection in the rat is still controversial. Whereas recent studies have not found the presence of active caspase-3 and caspase-9 indicating that these caspases are not involved in apoptosis of nigral DA neurons [29], [30], other studies suggest that activation of caspase-3 participates in programmed cell death of nigral neurons [26], [31], [32]. This controversy is further strengthened by the recent findings that caspase-3 also participates in nonapoptotic functions, such as activation of microglia [33], [34]. These antecedents suggest that cleaved caspase-3 might be associated with apoptosis of DA neurons and neuroinflammation, but in different stages of the neurodegenerative process.

Glycogen synthase kinase (GSK)-3β is implicated in the signaling pathway of neuronal apoptosis activated by oxidative stress [35], a central factor in the neuropathological process of PD [18], [36]. The GSK-3β is activated by phosphorylation of the tyrosine 216 residue (Y216) located in the kinase domain and inactivated by phosphorylation of the amino-terminal serine 9 residue (S9) [37]–[40]. Recent studies made in SH-SY5Y cells, a human-DA cell line, suggest that GSK-3β plays a pivotal role in the intracellular signaling pathways involved in DA cell death activated by 6-OHDA [41], [42]. A similar conclusion was achieved in the MPTP mouse model of PD [43]. To date, it is unknown whether the GSK-3β pY216 in the SNc is activated by the striatal injection of 6-OHDA in the rat.

Our work evaluated whether a single striatal injection of 6-OHDA causes apoptosis and activation of caspase-3 and GSK-3β pY216 in DA neurons. We documented the loss of DA neurons using three neuron markers; TH, the rate-limiting enzyme in dopamine synthesis, NeuN, a neuronal nuclear antigen, and β-III tubulin, a neuronal cytoskeleton protein. Apoptosis activation was determined using Apostain, a specific marker of apoptosis [24], [44], [45], and immunostaining of cleaved caspase-3 and GSK-3β pY216. In addition, the possible role of cleaved caspase-3 in neuroinflammation was evaluated by double immunofluorescence against cleaved caspase-3 and OX42, a microglia marker [46], or glial fibrillary acidic protein (GFAP), an astrocyte marker [47]. Our results contribute to the knowledge of the mechanism of 6-OHDA cytotoxicity in the rat.

## Materials and Methods

### Ethics Statement

All procedures were in accordance with the current Mexican legislation, NOM-062-ZOO-1999 (SAGARPA), based on the Guide for the Care and Use of Laboratory Animals, NRC. The CINVESTAV Institutional Animal Care and Use Committee approved our procedures for animal use (protocol # 109-02). All efforts were made to minimize animal suffering.

### Animals

Adult male Wistar rats (220–230 g body weight) bred in our facilities were maintained under constant room temperature (RT; 23°C), 12 h −12 h light-dark cycle, with food and water *ad libitum*. The rats were anesthetized with a single dose of ketamine (100 mg/kg) and xilazine (10 mg/kg; ip) for all surgical procedures.

### Stereotaxic Surgery

Anesthetized rats were fixed in a stereotaxic apparatus (Stoelting; Wood Dale ILL, USA) to trepan their skulls with a dental drill at the appropriate location. A single dose (3 µL) of 6-OHDA (Sigma-Aldrich; St. Louis, MO) solution (6.8 µg free base/µL in phosphate-buffered saline, pH 7.4 (PBS), containing 0.02% ascorbic acid) was injected into the left neostriatum at the coordinates AP, +6.9 mm from the interaural midpoint; ML, +4 mm from the intraparietal suture; DV, −5.5 mm from duramater. The control of the apoptotic process was a group of animals injected with 2 µL of a 50 nM staurosporine (Sigma-Aldrich; St. Louis, MO, USA) solution into the SNc at the coordinates AP +2.4 mm from the interaural midpoint; ML +1.8 mm from the intraparietal suture; DV −6.9 mm from duramater. The flow rate of 6-OHDA and staurosporine injections were maintained by a micropump Mod. 100 (Stoelting; Wood Dale, ILL, USA) at 0.2 µL/min. After the total dose was injected into each site, the needle was allowed to remain in the brain for 10 min.

### Immunostaining Techniques

The rats were deeply anesthetized with sodium pentobarbital (50 mg/kg ip) and perfused through the ascending aorta with 30 mL of PBS, followed by 100 mL of 4% paraformaldehyde in PBS. The brain was then removed and maintained in the fixative for 48 h at 4°C. After an overnight incubation in PBS containing 30% sucrose at 4°C, the brain was frozen and sectioned into 35-µm or 15-µm (depending on the immunostaining protocol) slices on the coronal plane using a freezing sliding microtome (Jung Histoslide 2000R; Leica; Heidelberg, Germany). A total of 50 to 55 slices *per* rat containing the whole SNc and 5 representative slices of the neostriatum *per* rat were collected in PBS. To obtain a representative sample of the SNc, the slices were consecutively distributed into 5 wells, with each well then containing 10 to 11 serial slices. After permeabilization with PBS-0.1% Triton, the nonspecific binding sites were blocked with 10% horse serum (Invitrogen; Carlsbad, CA, USA) in PBS-0.3% Triton-X-100 for 1 h [3], [7]. TH-immunohistochemistry was made after depletion of endogenous peroxidase using PBS-Triton-X-100 0.3% solution containing 3% hydrogen peroxide and 10% methanol at RT. The primary antibody was a mouse monoclonal anti-TH clone TH-2 (1∶1000; Sigma-Aldrich; St. Louis, MO, USA) and the secondary antibody was a horse biotinylated anti-mouse IgG (H+L) (1∶200; Vector Laboratories; Burlingame, CA, USA). The immunohistochemical staining was developed using the avidin-biotin-peroxidase complex (1∶10; ABC Kit; Vector Laboratories; Burlingame, CA, USA) and 3′3-diaminobenzidine (DAB; Sigma-Aldrich; St. Louis, MO, USA). Immunohistochemistry with a mouse monoclonal anti-ssDNA (Apostain; 1∶10; Bender Biosystems; Vienna, Austria) was used to detect condensed chromatin of apoptotic cells in 15-µm mesencephalon slices mounted on glass slides previously treated with poly-L-lysine [44], [45]. The secondary antibody was a peroxidase-conjugated rat monoclonal anti-mouse IgM (1∶100; Zymed; San Francisco, CA, USA).The immunohistochemical staining was observed with a Leica DMIRE2 microscope with a 20x objective and images were digitalized with a DC300F camera (Leica; Nussloch, Germany). The counting of the TH(+) neurons of the SNc and ventral tegmental area (VTA) was done on one out of every five TH-immunostaining slices (total slices = 10 *per* rat; *n* = 3 rats *per* group). Digital images of the SNc and VTA were projected onto a screen monitor for neuron counting.

For double immunofluorescence assays, the primary antibodies were mouse monoclonal anti-TH clone TH-2 (1∶1000; Sigma-Aldrich; St. Louis, MO, USA), mouse monoclonal anti-NeuN (1∶500; Cell Signaling; Danvers, MA, USA), mouse monoclonal anti-GSK-3β pY216 (1∶600; BD Transduction Laboratories; Franklin Lakes, NJ, USA), mouse monoclonal anti-GFAP (1∶250; Cell Signaling; Danvers, MA, USA), mouse monoclonal anti-OX42 (1∶200; Abcam; Cambridge, UK), rabbit polyclonal anti-TH (1∶1000; Chemicon Inc.; Billerica, MA, USA), rabbit polyclonal anti-cleaved-caspase-3-Asp 175 (1∶300; Cell Signaling; Danvers, MA, USA), and rabbit polyclonal anti-β-III tubulin (1∶300; Sigma-Aldrich; St. Louis, MO, USA). The secondary antibodies were fluorescein isothiocyanate (FITC) sheep anti-mouse H+L IgG (1∶60; Jackson Immunoresearch; West Grove, PA, USA), Texas red horse anti-mouse H+L IgG (1∶300; Vector laboratories; Burlingame, CA, USA), FITC goat anti-rabbit H+L IgG (1∶60; Jackson Immunoresearch; West Grove, PA, USA), Texas red goat anti-rabbit H+L IgG (1∶300; Vector laboratories; Burlingame, CA, USA), and Alexa 488 chicken anti-mouse (1∶200; Invitrogen Molecular Probes; Eugene, Oregon, USA). For acquisition of images of double immunofluorescence against TH and NeuN, we used a Leica DMIRE2 microscope using the 5x and 40x objectives, and the filters K3 for FITC (green fluorescence) and TX2 for Texas (red fluorescence). The images were digitized with a Leica DC300F camera (Nussloch, Germany). A multispectral confocal-laser scanning microscope (TCS SPE, Leica; Heidelberg, Germany) was used to analyze the double immunofluorescence against TH and β-III tubulin, and TH, GFAP, or OX42 with cleaved-caspase-3. The fluorescence was viewed through 40x and 63x oil-immersion objectives at excitation-emission wavelengths of 405–465 nm (blue channel), 488–522 nm (green channel), and 568–635 nm (red channel). Ten to twenty consecutive 1-µm optical sections were obtained in the z-series (scanning rate 600 Hz). The images were processed by LAS AF software (Leica Microsystems; Nussloch, Germany). Negative controls were obtained by omitting the primary antibody and replacing it with an irrelevant antibody of the same IgG subclass.

### Western Blot Analysis

Western blotting was done in homogenates from nigra tissues as we previously described [7]. Proteins (50 µg) were subjected to electrophoresis in 12% SDS-PAGE gels and transferred onto PVDF membranes (Bio-Rad Laboratories; Hercules, CA, USA) to be independently immunolabeled. The primary antibodies were those for immunofluorescence studies, i.e. the mouse monoclonal anti-TH clone TH-2 (1∶1000), rabbit polyclonal anti-TH (1∶5000), rabbit polyclonal anti-cleaved-caspase-3-Asp 175 (1∶2500), rabbit polyclonal anti-β-III tubulin (1∶300), mouse monoclonal anti-GSK-3β, or mouse monoclonal anti-GSK-3β pY216 (1∶600). After washing, the PVDF membranes were incubated for 1 h with a suitable secondary antibody conjugated with horse radish peroxidase (HRP), either a goat anti-mouse IgG or a donkey anti-rabbit F(Ab)2 (1∶5000; Zymed, Cambridge, MA, USA). To normalize the total amount of protein per lane, membranes were stripped and incubated with a monoclonal mouse antibody against actin (1∶500; CINVESTAV, Mexico) followed with a HRP-conjugated goat anti-mouse IgG (1∶6000; Zymed; San Francisco, CA, USA).

### Statistical Analysis

All values are the mean values ± SEM. The comparisons between groups were made using a one-way analysis of variance (ANOVA), followed by the Newman-Keuls *post-hoc* test. The accepted significance was at *P*<0.05 and *P*<0.001.

## Results

### Loss of Dopamine Cells

**Figure 1:**
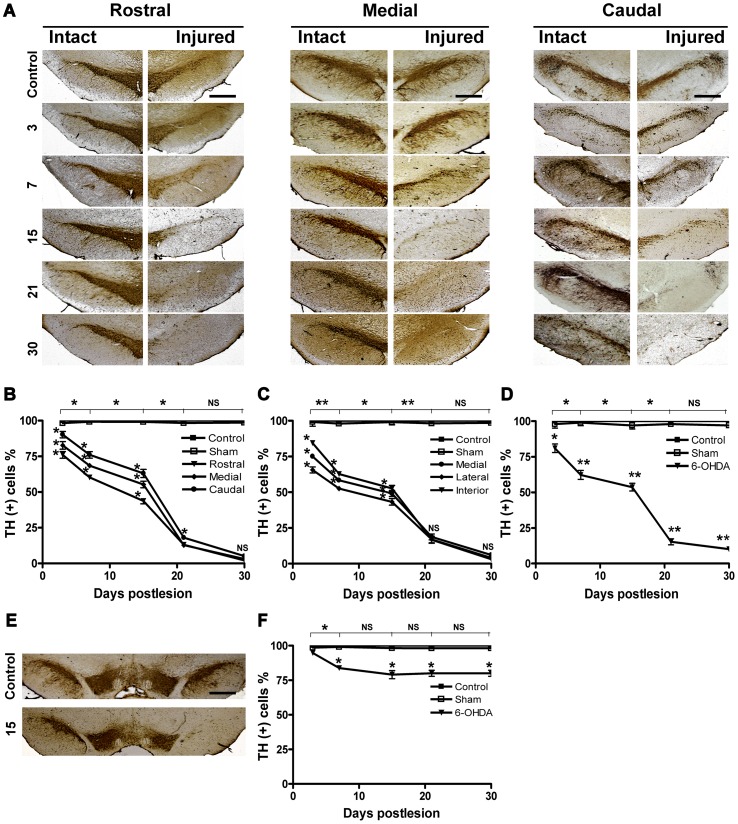
Progressive loss of TH(+) cells in the SNc after a 6-OHDA injection into the neostriatum. The cell counting was done on 10 to 11 coronal slices (35 µm) *per* rat (*n* = 3 per group). In panel A, representative micrographs of TH-immunostained slices taken from the rostral, medial, and caudal mesencephalon. In graphs B and C, cell counting in rostrocaudal direction and lateromedial direction respectively. In D, total counts of residual TH(+) cells. In E and F, representative micrograph showing TH immunostaining and cell counting in the VTA. The primary antibody was a mouse monoclonal anti-TH clone TH-2 and the secondary antibody was a horse biotinylated anti-mouse IgG (H+L). All values are expressed as the mean ± SEM *P<0.05, **P<0.001 when compared with the intact group or between groups; one-way ANOVA test and Newman-Keuls *post-hoc* test. NS = not significant. The scale bars = 1 mm in the first row (control) are common for all micrographs.

A single injection of 6-OHDA into the neostriatum mainly decreased the number of TH-immunoreactive cells in the SNc (Fig. 1A–D) and in a lesser proportion in the VTA (Fig. 1E–F). A 25% significant decrease in TH(+) cells was measured as early as three days postlesion in the three regions of the SNc studied (rostral, medial, and caudal) as compared with those of the intact control side (Fig. 1A–D). The loss of TH(+) cells progressively continued up to 30 days postlesion and was 90% as compared with the intact control (Fig. 1A,D). The loss of TH(+) cells progressed mainly in the rostrocaudal direction (Fig. 1B) and in the lateromedial direction (Fig. 1C). When the comparison between regions (rostral, medial, and caudal, or lateral, medial, and interior) was made, the counts of TH(+) cells showed a statistically significant difference from 3 to 15 days postlesion (Fig. 1A–C). The population of TH(+) cells in the VTA decreased 20% at day 7 and remained at that same percentage to the end of the study (Fig. 1E,F). The decrease in TH immunoreactivity caused by 6-OHDA in the neostriatum appeared earlier than in the substantia nigra (at day 3 postlesion) because of the close proximity with the site of the neurotoxin injection (Fig. S1).

**Figure 2:**
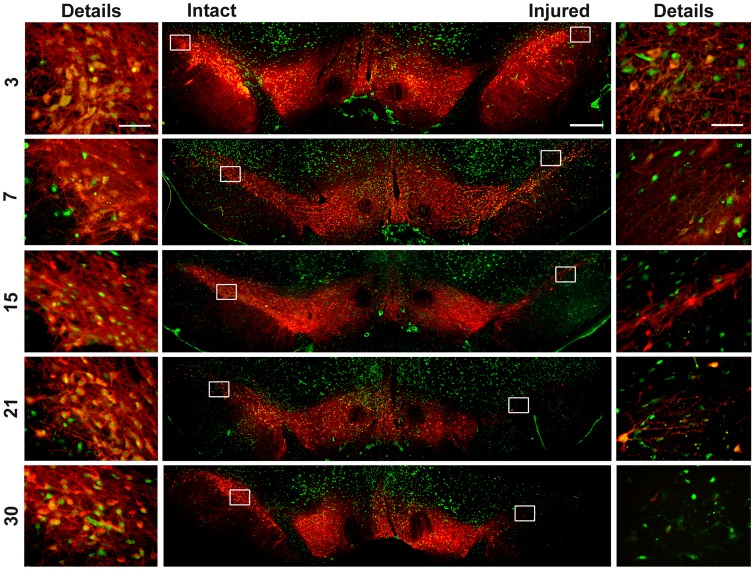
Representative merged micrographs of mesencephalon immunostained against NeuN and TH. The numbers at the left margin of the figures are the days after 6-OHDA lesion. The primary antibodies were a mouse monoclonal anti-NeuN and a rabbit polyclonal anti-TH. The secondary antibodies were sheep anti-mouse IgG (H+L) FITC conjugated and goat anti-rabbit IgG (H+L) Texas conjugated. The rectangles on the panoramic view show the regions that were amplified 40x (details). Scale bar = 1 mm (central panels) and 50 µm (details).

The studies of double immunofluorescence against TH and NeuN, a nuclear marker of neuronal lineage [48], showed that the loss of TH(+) cells was related to the disappearance of neuronal bodies after the 6-OHDA lesion. This was qualitatively shown in the medial region of the SNc. During the advance of a 6-OHDA lesion, there was a simultaneous loss of cells with TH and NeuN immunostaining (Fig. 2). In addition to cells with double immunostaining, cells with immunoreactivity only to NeuN are seen in the SNc on the days studied and remained until the end of the study.

### Loss of Dopamine Neuron Cytoskeleton

**Figure 3:**
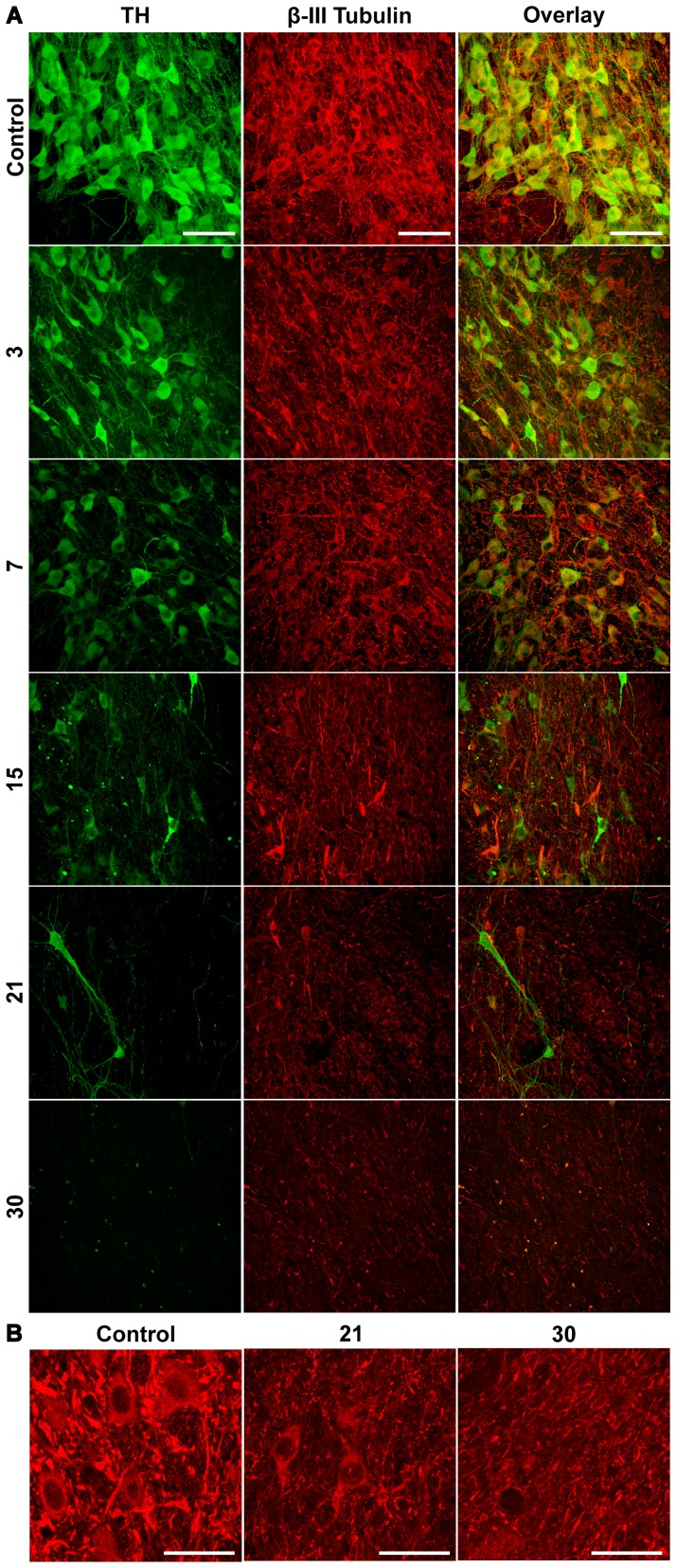
Neuronal cytoskeleton loss occurs along with the progressive loss of TH(+) cells in the SNc after the 6-OHDA injection into the neostriatum. In panel A, representative micrographs of mesencephalon with double immunostaining to β-III tubulin and TH. The numbers at the left margin of the figures show the days after the 6-OHDA lesion. The primary antibodies were a mouse anti-TH monoclonal and a rabbit anti-β-III tubulin polyclonal. The secondary antibodies were sheep anti-mouse IgG (H+L) FITC conjugated and goat anti-rabbit IgG (H+L) Texas conjugated. In panel B, representative micrographs of SNc showing details of the loss of β-tubulin III immunoreactivity at days 21 and 30 after the 6-OHDA injection as compared with the intact control side. Scale bars = 20 µm. The scale bars in the panel A are common for all the micrographs.

In the control condition, a great number of cells displayed immunostaining to TH and β-III tubulin (Fig. 3A). After the 6-OHDA intrastriatal injection, a decrease in immunostaining to β-III tubulin, a constitutive protein of the neuronal cytoskeleton [49], occurred, along with the loss of the TH(+) cells (Fig. 3A). After 15 days postlesion, some well-defined body cells with only β-III tubulin-immunostaining remain in the SNc (Fig. 3B). These results support the hypothesis that the 6-OHDA-caused neurodegeneration leads to death of dopamine cells in the SNc.

### Apoptosis Involvement in Dopamine Cell Death

**Figure 4:**
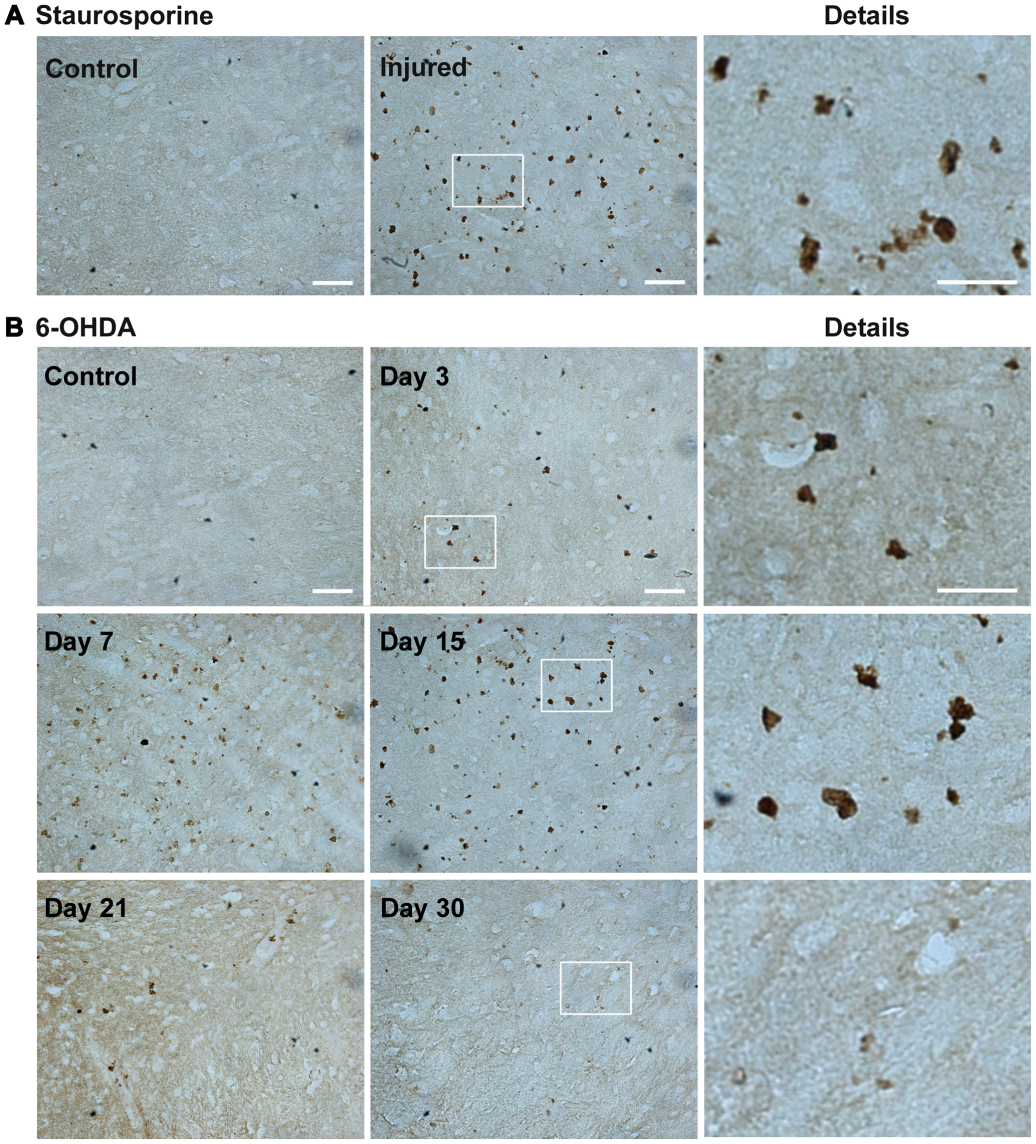
The injection of 6-OHDA into the neostriatum mainly leads to cell death via apoptosis in the SNc. In insert A, apoptosis caused by an intranigral injection of staurosporine (50 nM), positive control of the Apostain technique. In panel B, representative micrographs of SNc immunostained using the Apostain technique at different days after the 6-OHDA lesion. The micrographs of the details are the 63x amplification of the areas within the rectangles on the corresponding panoramic view. The primary antibody was a monoclonal mouse anti-ssDNA (Apostain). The secondary antibody was a peroxidase-conjugated rat monoclonal anti-mouse IgM. Scale bars = 50 µm (panoramic view) and 20 µm (details).

The participation of apoptosis in the 6-OHDA-caused neurodegeneration was supported by the results of Apostain assay. This assay detects single-stranded (ss)DNA in areas where DNA scaffolding was degraded by the action of caspases [44], [45]. Staurosporine, known to cause apoptosis, injected into the SNC was used as a positive control of the Apostain technique (Fig. 4A). Both 6-OHDA and staurosporine injection led to the appearance of Apostain(+) small rough bodies (2.5 to 6.3 µm), which were scattered or gathered together throughout the area of the SNc (Fig. 4). The amount of Apostain(+) bodies increased from day 3 to day 15 after the 6-OHDA lesion (Fig. 4B). A decrease of the amount Apostain(+) bodies was measured from day 21 to day 30 after the 6-OHDA lesion (Fig. 4B).

### Cellular Distribution of Active Caspase-3

**Figure 5:**
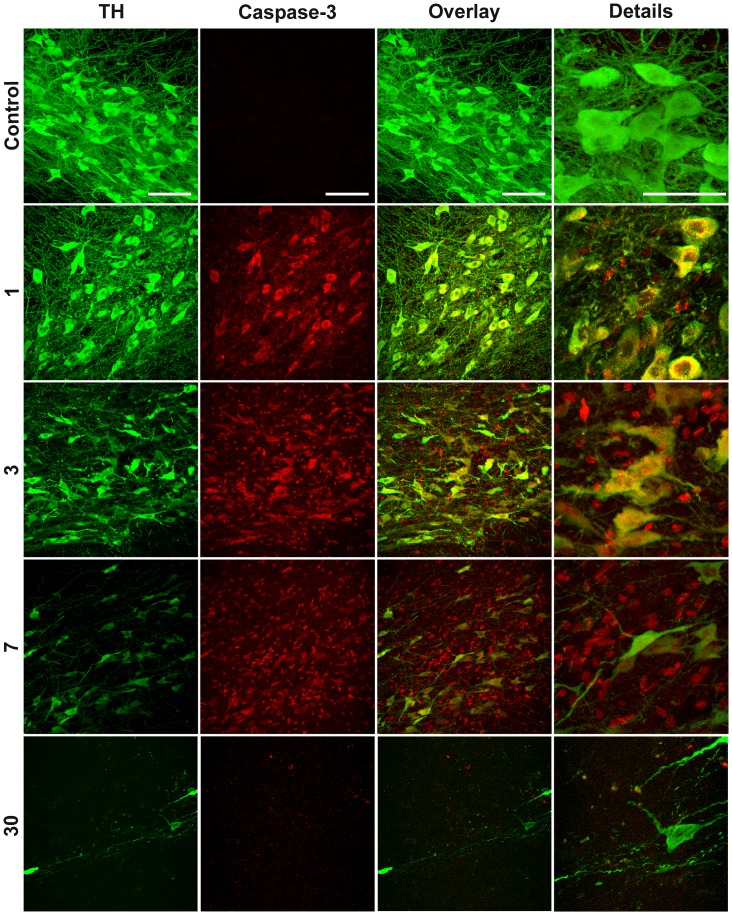
The injection of 6-OHDA into the neostriatum leads to the activation of caspase-3 in the SNc. Representative confocal micrographs of mesencephalon immunostained against TH and cleaved caspase-3. The numbers at the left margin of the figures are the days after the 6-OHDA lesion. The primary antibodies were a mouse monoclonal anti-TH and a rabbit polyclonal anti-cleaved-caspase-3. The secondary antibodies were FITC goat anti-mouse IgG (H+L) conjugated and Texas red goat anti-rabbit H+L IgG. The scale bars = 50 µm in the first row are common for all the micrographs.

**Figure 6:**
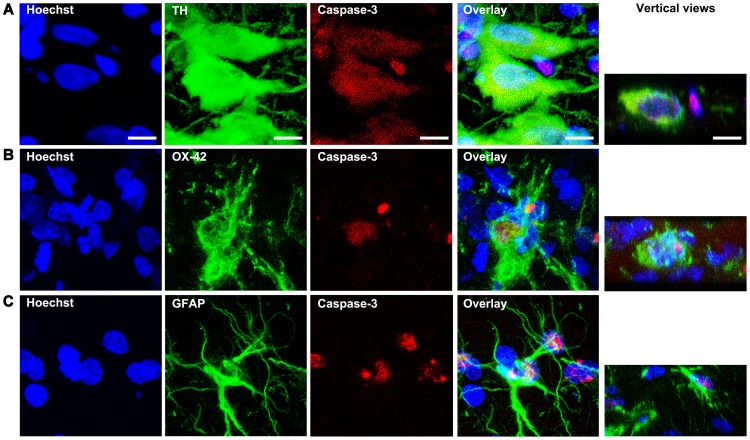
Active caspase-3 is present in microglia and astrocytes in the SNc at seven days after the 6-OHDA striatal injection. In panels A, B and C, representative confocal micrographs of mesencephalon immunostained against TH, OX42, GFAP, and cleaved caspase-3. The vertical views correspond to 1-µm optical sections in the vertical plane of the corresponding merged images. The primary antibodies were mouse monoclonal anti-TH, mouse monoclonal anti-OX42, mouse monoclonal anti-GFAP, and rabbit polyclonal anti-cleaved-caspase-3. The secondary antibodies were Alexa 488 chicken anti-mouse and Texas red goat anti-rabbit H+L IgG. Scale bars = 10 µm in the first row are common for all the micrographs.

The confocal microscopy analysis showed that cleaved caspase-3 immunostaining was absent in the SNc without a lesion and was present as early as day 1 postlesion (Fig. 5). The cleaved caspase-3-immunostaining reached the maximum intensity at day 7 postlesion and decreased thereafter but was present until the end of the study (Fig. 5). The cleaved caspase-3 immunoreactivity was collocated with most of TH(+) cells, showing a nuclear (day 3) and perinuclear (day 7) presence (Fig. 5 and Fig. 6A). A great number of small cells with only cleaved caspase-3 immunostaining were also seen scattered in the neuropil of the SNc after day 7 postlesion (Fig. 5 and Fig. 6A). To identify to what cell population would correspond the non-TH cleaved caspase-3 expression, we explored two glial populations, namely the OX42-positive microglia and GFAP-positive astrocytes at day 7 after the 6-OHDA lesion (Fig. 6B,C). An increase in the number of those glial cell populations was measured in the lesion side suggesting the presence of neuroinflammation (data not shown). The presence of cleaved caspase-3 immunostaining was observed in the cytoplasm and nucleus of both OX42-positive microglia (6B) and GFAP-positive astrocyte cells (6C).

### Activation of GSK-3β

**Figure 7:**
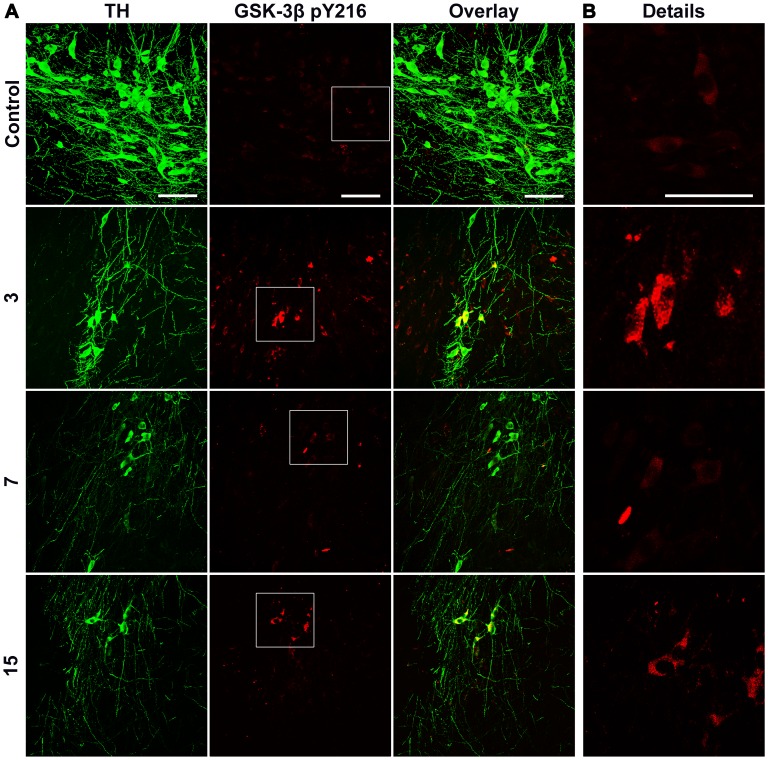
Increase of the GSK-3β pY216 immunoreactivity in the substantia nigra after a striatal 6-OHDA injection. In panel A, representative confocal micrographs of the SNc double immunostained against TH and GSK-3β pY216 at different days (shown at the left margin) after the 6-OHDA striatal injection. In B, the micrographs in the Details column correspond to amplification of the area within the corresponding rectangles on the panoramic view. The primary antibodies were a rabbit polyclonal anti-TH and a mouse monoclonal anti-GSK-3β pY216. The secondary antibodies were fluorescein isothiocyanate (FITC) goat anti-rabbit H+L IgG and Texas red horse anti-mouse H+L IgG. The scale bars = 50 µm of the first row are common for all the micrographs.

In our study, the phosphorylated form of GSK-3β pY216 in TH(+) cells was evaluated because this phosphorylation site reflects the activation of this kinase [39], [40], [50]. The confocal microscopy analysis of the control SNc with double immunostaining against TH and GSK-3β pY216 showed the presence of basal levels of GSK-3β pY216-immunostaining in both TH(+) and TH(−) cells (Fig. 7). After the 6-OHDA injection, the immunostaining of GSK-3β pY216 was more intense in the TH(+) cells at day 3 and 15 postlesion (Fig. 7A,B), thus suggesting two peaks of activation of GSK-3β.

### Time Course of Dopamine-cell Death Markers

**Figure 8:**
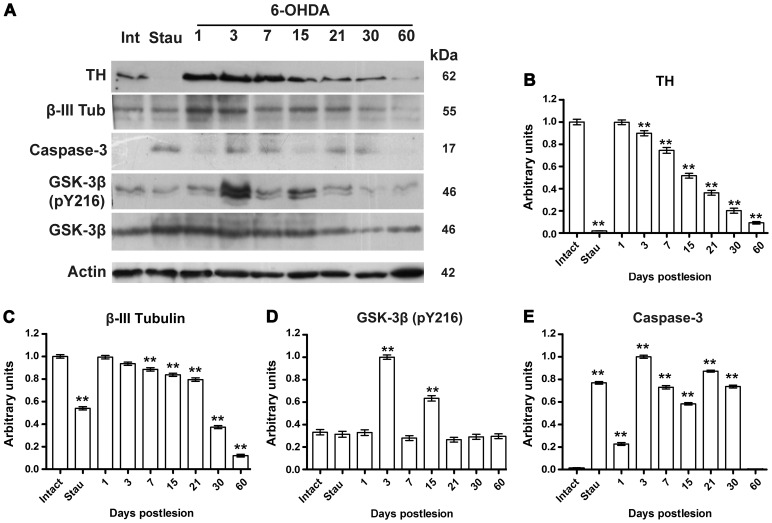
Changes in the protein levels in the SNc after a 6-OHDA injection into the ipsilateral striatum. In panel A, a representative photograph of a western blot analysis of the two markers of dopamine neurons (TH and β-III tubulin) and two markers of apoptotic process (cleaved caspase-3 and GSK3β pY216). The graphs B-E shows the densitometry analysis of the bands of the protein of interest (*n* = 3). The values (arbitrary unities) result from normalization of the ratio between the density of the band of interest and the density of the corresponding loading protein compared to the corresponding ratio in the intact control SNc. Actin was used to normalize TH, β-III tubulin, and cleaved caspase-3. The GSK3β protein was also used to normalize values to GSK3β pY216. The control groups were the corresponding contralateral sides of injection (intact). Intranigral injection of 50 nM staurosporine (Stau) was used as positive control of apoptosis evaluated 24-h after administration. Int = intact SNc. All values are the mean ± SEM. Differences of the groups were analyzed with a one-way ANOVA test and Newman-Keuls *post-hoc* test. **P<0.001 when compared with intact group.

Western blot was used as an independent technique to evaluate the two markers of dopamine neurons (TH and β-III tubulin) and two markers of the apoptotic process (cleaved caspase-3 and GSK3β pY216). GSK3β and actin were used as internal controls (Fig. 8A). Staurosporine (50 nM) injection into the SNc was the positive control of apoptosis (Fig. 8A). After the 6-OHDA injection, the levels of TH and β-III tubulin progressively decreased over time to reach a remaining level of 7% compared to the levels of the intact SNc at day 60 postlesion (Fig. 8A–C). The cleaved caspase-3 was not detected in the intact control SNc (Fig. 8A,E). The level of this protein increased at day 1 and reached the maximum value at day 3 after the 6-OHDA lesion. After decreasing to the days 7 and 15, a second increase in the level of cleaved caspase-3 occurred at day 21, thereafter it decreased to reach undetectable levels at day 60 postlesion (Fig. 8A,E). In agreement with the results of confocal microscopy (Fig. 7), basal levels of GSK3β pY216 were detected in the intact control SNc and for the two increments observed at days 3 and 15 after the 6-OHDA lesion (Fig. 8A,D).

## Discussion

This work shows for the first time that 6-OHDA injected into the neostriatum leads to a progressive loss of cytoskeleton integrity (decrease of β-III tubulin) of the TH(+) cells in the SNc. This finding together with the loss of TH- and NeuN-immunolabeled cells is sound evidence of the progressive death of the DA neurons. Along with this, our results also demonstrate activation of caspase-3 and GSK3β in DA neurons. Our finding that the presence of GSK3β pY216, the active form of this enzyme, in TH(+) cells after a 6-ODHA lesion suggests that the activation of caspase-3 might have resulted from activation of the intrinsic pathway of apoptosis [35], where the active GSK3β plays a pivotal role [40].

An increasing number of studies *in vitro* have demonstrated that GSK3β pY216, the active form of this enzyme, mediates the 6-OHDA-triggered neuronal death [38], [40]–[42], [51]. On this basis, the increased immunoreactivity of GSK3β pY216 found in this study in nigral DA neurons at days 3 and 15 shows activation of GSK3β pY216 and suggests participation in the 6-OHDA-triggered DA neuron death *in vivo*. Our results of the Western blot confirmed those two peaks of GSK-3β activation, of which at least the first peak coincides with the maximum peak of active caspase-3 suggesting that at this time of the 6-OHDA lesion the GSK3β activation mediates DA neuron death. However, a recent study suggests that 6-OHDA does not cause GSK3β activation because the increase in GSK3β S9, the inactive form of this enzyme, caused by a 16-day lithium treatment, did not prevent DA neuron degeneration at 72 h after a 6-OHDA injection in the SNc [52]. This apparent controversy can be explained by the possibility that the activity of GSK3β pY216 overcame the inhibitory effect of GSK3β S9. The GSK3β pY216 activation might have resulted from the decrease of TrkB activation followed by the decrease of the inhibitory effect of Akt [38], [41], [53]–[55]. In turn, the GSK3β pY216 can account for the activation of the mitochondrial-death pathway (intrinsic pathway) [35], which results in increased cytochrome c release from the mitochondria [56], [57] and activation of caspase-9 and −3 in the 6-OHDA PD rat model [26], [28]. Supporting the participation of the intrinsic death pathway, a recent work in mice showed that 6-OHDA causes the release of cytochrome c and DA cell death as a consequence of the upregulation of PUMA and activation of p53 [58]–[60]. According to this, the treatment with z-DIPD-fmk or zDEVD-fmk, specific inhibitors of caspase-3 activation, prevent the caspase-3 dependent TH+ cell death caused by 6-OHDA both *in vitro* and *in vivo* [61]–[64]. In contrast, Kim et al. [30] demonstrate atrophy and progressive death of DA neurons with evidence of nuclear translocation of apoptosis-inducing factor (AIF) in wild-type mice after a striatal injection of 6-OHDA, without activation of caspase-3, cytoplasmic release of cytochrome C or signs of apoptosis [30]. The authors also show that 6-OHDA-induced DA neuron death is mediated by Bax-dependent AIF activation, because the genetic deletion of the proapoptotic gene Bax completely prevented DA neuron death and the nuclear translocation of AIF [30]. This apparent controversy can be explained by the different time of evaluation of the apoptotic markers. Kim et al. [30] explored the caspase-3 activation and cytochrome C release after 2 and 6 weeks postlesion, whereas others evaluated those apoptotic markers in the early stage of the neurodegeneration (1–7 days) [26], [27], [31], [63]. Nevertheless, those works reflect the variability of pathways that can converge into the death of DA neurons in the 6-OHDA animal model.

In our work, the Apostain technique was used to show apoptosis in DA neurons after a 6-OHDA intrastriatal injection. Studies using TUNEL and DNA laddering had suggested that 6-OHDA also injected into the neostriatum led to apoptosis of nigral DA neurons [20], [22], [31], [65], [66]. Apostain detects single-stranded DNA in condensed chromatin, which is a definite marker of apoptosis [44], [45]. Unlike Apostain, TUNEL is not specific for apoptosis because it also recognizes fragments of double-stranded DNA that are present in necrotic cells [23], [24]. Nevertheless, the results of the TUNEL and DNA laddering assay show that the nonrandom internucleosomal fragmentation of DNA occurs in the 6-OHDA rat model [65]–[68]. These data strongly suggest the participation of caspase-3, which activates the enzyme responsible for apoptotic DNA fragmentation (Caspase-Activated DNase) [69], [70]. Our immunofluorescence results showed that cleaved caspase-3 immunoreactivity collocated with TH(+) cells. Based on these results, we can propose that active caspase-3 plays a crucial role in the 6-OHDA-activated apoptosis of DA cells. The active caspase-3 can also play a role in neuroinflammation caused by 6-OHDA [46], [71], because our results with double immunofluorescence showed collocation of caspase-3 immunoreactivity with OX42(+) and GFAP (+) cells after day 3 postlesion. This finding supports the new role of active caspase-3 in the control of microglia and brain inflammation [72], [73]. Microglia can be neuroprotective, for example, by degrading cell debris or misfolded proteins [74]. However, a recent, new line of evidence points out that activated microglial cells are likely to be neurotoxic because they release proinflammatory cytokines like the tumor necrosis factor-α (TNF-α) and IL-1beta causing further tissue damage [75], [76].

We also showed that the TH(+) cell loss progresses from lateromedial and rostrocaudal directions in agreement with a previous report [1], which only described that 6-OHDA affect the rostral portion of SNc. At 30 days postlesion, our results showed 10% of residual TH(+) cells in the SNc, which could correspond to the subtype calretinin positive (CR +) [77], [78] or calbindin positive (CB+) [79]–[81] DA neurons that are resistant to the toxic action of 6-OHDA. In addition, there were TH(−) cells with immunoreactivity to NeuN or β-III tubulin suggesting that these cells could be GABAergic neurons that constitute 5% of the nigral cell population [82].

In conclusion, our results showed that 6-OHDA injected into the neostriatum leads to a progressive apoptosis of nigral DA neurons and activation of GSK3 β pY216 and caspase-3. The presence of active caspase-3 in glial cells supports its participation in a neuroinflammatory process occurring in the SNc. The presence of GSK3β pY216 suggests that the activation of caspase-3 might have resulted from activation of the intrinsic pathway of apoptosis. The time-course study showed that the critical period of apoptosis occurs between days 7 and 15 postlesion. Our results provide new insight into the mechanism of 6-OHDA cytotoxicity, which suitably models the processes of neurodegeneration and neuroinflammation caused by oxidative stress in PD [11]. This knowledge will be useful for the evaluation of promising therapeutic interventions and the understanding of their mechanisms in the 6-OHDA model.

## Supporting Information

Figure S1 **Loss of TH(+) cells after a single injection of 6-OHDA in the neostriatum.** Representative micrographs of TH-immunostained slices taken from the medial neostriatum. The primary antibody was a mouse monoclonal anti-TH clone TH-2 and the secondary antibody was a horse biotinylated anti-mouse IgG (H+L). The scale bar = 1 mm in the first row (control) is common for all micrographs.
(TIF)
