# Supplementary figures and images for: Activation of GSK-3β and Caspase-3 Occurs in Nigral Dopamine Neurons during the Development of Apoptosis Activated by a Striatal Injection of 6-Hydroxydopamine
Source: PLoS One. 2013 Aug 5;8(8):e70951. doi: 10.1371/journal.pone.0070951 (PMC3733721; doi:10.1371/journal.pone.0070951)

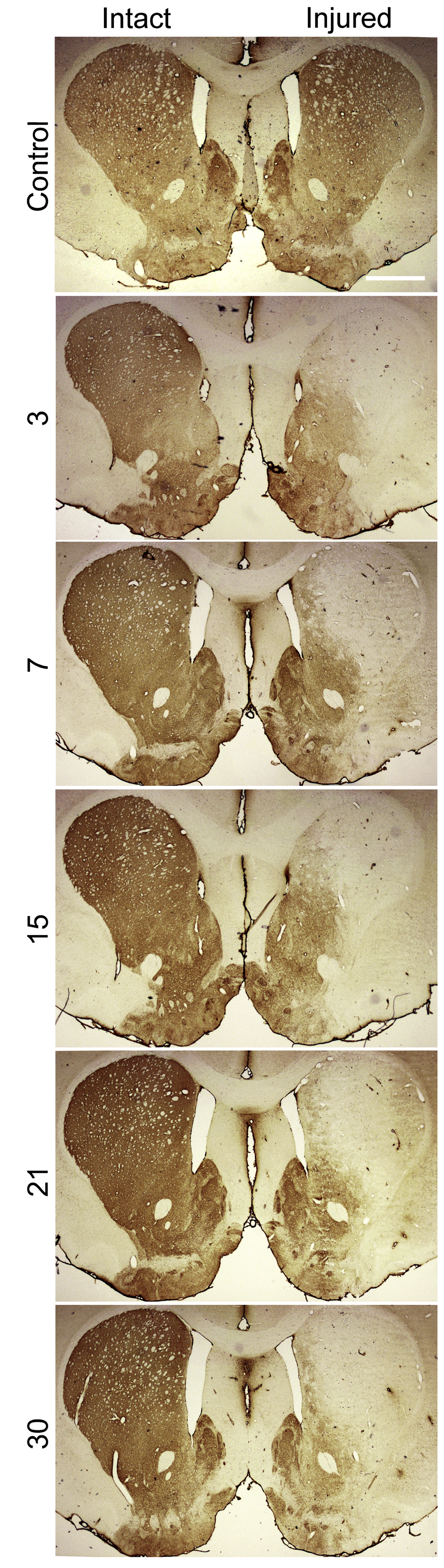

Supplement: Figure S1 — Loss of TH(+) cells after a single injection of 6-OHDA in the neostriatum. Representative micrographs of TH-immunostained slices taken from the medial neostriatum. The primary antibody was a mouse monoclonal anti-TH clone TH-2 and the secondary antibody was a horse biotinylated anti-mouse IgG (H+L). The scale bar = 1 mm in the first row (control) is common for all micrographs. (TIF) [file pone.0070951.s001.tif]
